# Tailoring mHealth for Healthy Aging: Focus Group Study With Retirement-Age Adults

**DOI:** 10.2196/70051

**Published:** 2025-12-15

**Authors:** Paula Collazo-Castiñeira, Rocío Rodríguez-Rey, Alfonso J Cruz-Jentoft, Somaya Ben Allouch, Doris Eglseer, Josje Schoufour, Eva Topinková, Peter J M Weijs, Yves Boirie, Macarena Sánchez-Izquierdo

**Affiliations:** 1 Department of Psychology Comillas Pontifical University Madrid Spain; 2 Servicio de Geriatría Hospital Universitario Ramón y Cajal (Instituto Ramón y Cajal de Investigación Sanitaria) Madrid Spain; 3 UNINPSI Clinical Psychology Center Madrid Spain; 4 Informatics Institute University of Amsterdam Amsterdam The Netherlands; 5 Digital Life Amsterdam University of Applied Sciences Amsterdam The Netherlands; 6 Institute of Nursing Science Medical University of Graz Graz Austria; 7 Department of Nutrition and Dietetics Faculty of Health, Sport and Physical Activity Amsterdam University of Applied Sciences Amsterdam The Netherlands; 8 Department of Geriatrics and Internal Medicine First Faculty of Medicine Charles University Prague Czech Republic; 9 Department of Nutrition and Dietetics Amsterdam Public Health Institute Amsterdam University Medical Centers Amsterdam The Netherlands; 10 Clinical Nutrition Department University Hospital Centre Université Clermont Auvergne Clermont-Ferrand France

**Keywords:** mHealth, eHealth, digital health, older adults, retirement, technology acceptance, behavior change techniques, persuasive design, social support, digital literacy, telemedicine

## Abstract

**Background:**

The adoption of mobile health (mHealth) technologies among older adults remains significantly lower than in younger populations, despite their potential to promote healthier lifestyles and mitigate age-related health risks.

**Objective:**

This study aims to explore the perspectives of retirement-age adults on mHealth interventions, identifying factors that influence their adoption, such as persuasive elements in the app design and psychological techniques.

**Methods:**

A qualitative focus group study was conducted with 19 Spanish participants recruited from urban community settings in Madrid, Spain (mean age 61.5 years; 15/19, 79% women). Participants discussed their attitudes, barriers, and preferences for mHealth tools. Focus groups were recorded, transcribed, and coded using an iterative process to ensure rigorous data analysis. An abductive approach was followed, using the persuasive design principles framework and the behavior change techniques’ taxonomy, and representing any theme outside those frameworks.

**Results:**

Participants expressed generally positive attitudes toward mHealth tools, favoring intuitive, user-friendly designs that are minimally time-demanding. However, significant barriers also emerged, such as low digital literacy and concerns about technology dependence. Key design preferences (persuasive design principles) and psychological techniques (behavior change techniques) were deemed beneficial, with preferred features such as tailored and meaningful goal-setting, self-monitoring, positive feedback (eg, congratulating messages after achieving a goal; social rewards), and a moderated use of notifications and prompts. Participants also stressed the importance of age-appropriate recommendations (eg, suggested diets for their age and characteristics) and design (eg, accessible, easy-to-use interfaces and human-like communication). Additionally, some preferences appeared to be culturally grounded (eg, rejection of anglicisms and the desire for locally relevant content, such as suggested activities specific to Madrid). Social support mechanisms, such as group activities and peer interactions through mHealth, were seen as critical for fostering motivation and engagement.

**Conclusions:**

mHealth interventions for this population should offer accessible and easy-to-use interfaces along with initial tutorials, facilitating an easy onboarding to overcome low digital literacy, thereby enhancing both usability and initial adoption. Furthermore, by providing meaningful, tailored content (eg, personalized diets and goals) and social features that foster peer connection (eg, user chats or organized activities), these tools may better support sustained engagement over time.

## Introduction

Chronic conditions, such as cardiovascular diseases, diabetes, and hypertension, affect nearly a third of the adult population, and this prevalence progressively increases with age, significantly reducing individuals’ quality of life and life expectancy [1-3]. Research consistently highlights that maintaining healthy behaviors, such as engaging in regular physical activity and a balanced diet, has protective effects against these conditions [4,5], increasing up to 10 years of living without chronic conditions [6]. While retirement-age adults (50-70 years old [7-10]) are often motivated to adopt positive lifestyle changes, evidence suggests a decline in health-related behaviors during this period [11,12]. Therefore, this period offers a strategic opportunity for targeted interventions.

A promising approach for promoting such behaviors would be through eHealth formats, understood as health services delivered via electronic devices, and specifically mobile health (mHealth) formats, delivered via mobile devices [13]. These tools can reach a broad audience while obtaining cost-effective positive results [14]. However, meta-analyses assessing the effectiveness of eHealth interventions targeting older adult users (typically aged 50 years and older) reveal inconsistent results, with limited confidence in the available literature [15]. Adoption of these tools among this population remains significantly lower than among younger adults across countries [16,17]. In Spain, this digital gap persists, with Internet usage declining by up to 28% as age increases [18], and less than 20% of older adults frequently use health-related technology in Spain [19].

This disparity is largely attributed to designs that fail to consider older users’ specific needs and preferences [20,21]. Age-related cognitive, psychological, motor, and sensory changes impact how older adults interact with technology, differentiating their needs from those of younger users [20,22]. Barriers such as low self-efficacy, negative attitudes toward technology, and perceived difficulties in usability are prevalent [23]. Other challenges include visual impairments, motor control difficulties (eg, typing), and generation gaps (difficulty understanding terminology used by younger users) [21]. Given these challenges, research calls for incorporating older users’ perspectives into the design of eHealth and mHealth interventions [21].

This study aims to explore what retirement-age adults find persuasive in mHealth interventions and identify the factors influencing their use. This analysis builds upon a previous qualitative study conducted with the same sample, which focused on identifying their goals, motivations, and perceived facilitators and barriers related to the adoption and maintenance of health behaviors [24]. While the earlier analysis focused on general lifestyle change, this study investigates user perspectives specifically related to mHealth, with the aim of identifying persuasive strategies and preferred features that may enhance adoption, usability, and engagement. To the best of our knowledge, this is the first study conducted in Spain targeting this population to assess these aspects. Recent literature underscores the importance of understanding these factors within this population [25,26].

## Methods

This study followed a qualitative methodology using focus groups.

### Participants

The study focused on individuals aged 50 to 70 years living in Madrid, Spain. Age was the only formal inclusion criterion. Additionally, participants were required to be able to understand and communicate in Spanish, to participate meaningfully in a focus group discussion, and to attend the in-person sessions in Madrid (although place of residence was not an exclusion factor). This age range was chosen to reflect a broad spectrum of retirement experiences, encompassing those anticipating retirement, undergoing the transition, or already retired. This age range is consistent with the variability of retirement transitions in Spain [10]. Moreover, this range aligns with the age group commonly used in studies exploring retirement transition [7-9].

Recruitment was conducted using multiple community-based strategies to ensure diversity in age and socioeconomic background. Recruitment materials included a poster displayed in a public hospital (Hospital Universitario Ramón y Cajal) and a private psychology clinic (UNINPSI), as well as institutional emails distributed to staff members at both centers. The study was also disseminated digitally via social media platforms (eg, LinkedIn, WhatsApp) following a snowball approach. Finally, we proactively contacted 53 public community associations and senior day centers serving retirees and older adults to expand outreach beyond clinical and academic settings.

Out of 34 individuals who initially expressed interest in participating, 4 individuals were excluded for not meeting the minimum age requirement of 50 years. Invitations were extended to the remaining 30 eligible individuals, with 19 ultimately participating in the focus groups (Table 1). Nonparticipation reasons included nonresponses or failure to confirm attendance (n=5), scheduling conflicts (n=5), and one participant being unable to attend due to a COVID-19 diagnosis at the time of the focus group session.

**Table 1:** Demographics, app use (type of app), and digital literacy of focus group retirement-age participants (Madrid, Spain; 2022).

ID	Group	Job status	Profession	Current use of apps (type)	Digital literacy
P1	1	Retired	Homemaker	No	Low
P2	1	Working	Health care	Yes (health)	High
P3	1	Retired	Health care	X^a^	X
P4	1	Retired	Administration	X	X
P5	1	Retired	Administration	Yes (health)	X
P6	2	Working	Transport	X	X
P7	2	Working	Administration	Yes (health)	High
P8	2	Working	Domestic work	Yes (learning)	X
P9	2	Working	Social work	No	X
P10	2	Working	Security	Yes (X)^b^	X
P11	2	Working	Health care	X	X
P12	3	Working	Administration	Yes (bank)	High
P13	3	Working	Health care	Yes (X)	Low
P14	3	Retired	Health care	No	X
P15	3	Working	Administration	X	X
P16	3	Working	Health care	X	X
P17	4	Working	Health care	Yes (health)	Low
P18	4	Working	Research	No	Low
P19	4	Working	Health care	Yes (health)	X

^a^“X” indicates that the participant did not provide specific information for that category during the focus groups.

^b^In entries such as “Yes (X),” participants reported using apps but did not specify the type.

### Procedure

The focus groups were designed and moderated by 2 female clinical psychologists: a senior researcher with expertise in gerontology (MSI, Ms, PhD) and a PhD student (PCC, Ms). There was no preexisting relationship between the moderators and the participants, who were informed only of the interviewers’ professional roles and institutional affiliations. Before the sessions, the study’s purpose was clearly explained to all participants.

The focus groups were scheduled based on participant availability and preferred locations. Four focus groups were conducted in March 2022 at the Hospital Universitario Ramón y Cajal and UNINPSI Clinical Psychology Center (Table 1). Consistent with prior research, 4 groups are generally considered sufficient to achieve data saturation [27]; however, this was not formally tested in this study and therefore cannot be confirmed. Each session was held in a room with natural light, ventilation, and seating arranged in a circular format to facilitate open interaction among participants.

### Data Collection

A structured focus group discussion guide was used to guide the focus groups (Table S1 in Multimedia Appendix 1). The guide was designed to explore 2 distinct yet interconnected research objectives. The first section of the guide focused on identifying facilitators and barriers to adopting and maintaining health behaviors during retirement, findings that have been discussed in a separate publication [24]. This paper focused on the second section, which examined participants’ perspectives and usage of digital tools and mHealth. The number of questions was intentionally kept low and formulated in an open format to encourage participant interaction and debate (eg, Do/would you like using health apps?; Table S1 in Multimedia Appendix 1). Consent to record the audio was obtained from all participants prior to the sessions. Participants were not involved in reviewing the transcriptions or final results. Field notes were also taken to capture nonverbal interactions and contextual details. Each focus group lasted between 90 and 120 minutes.

### Data Analysis

**Figure 1:**
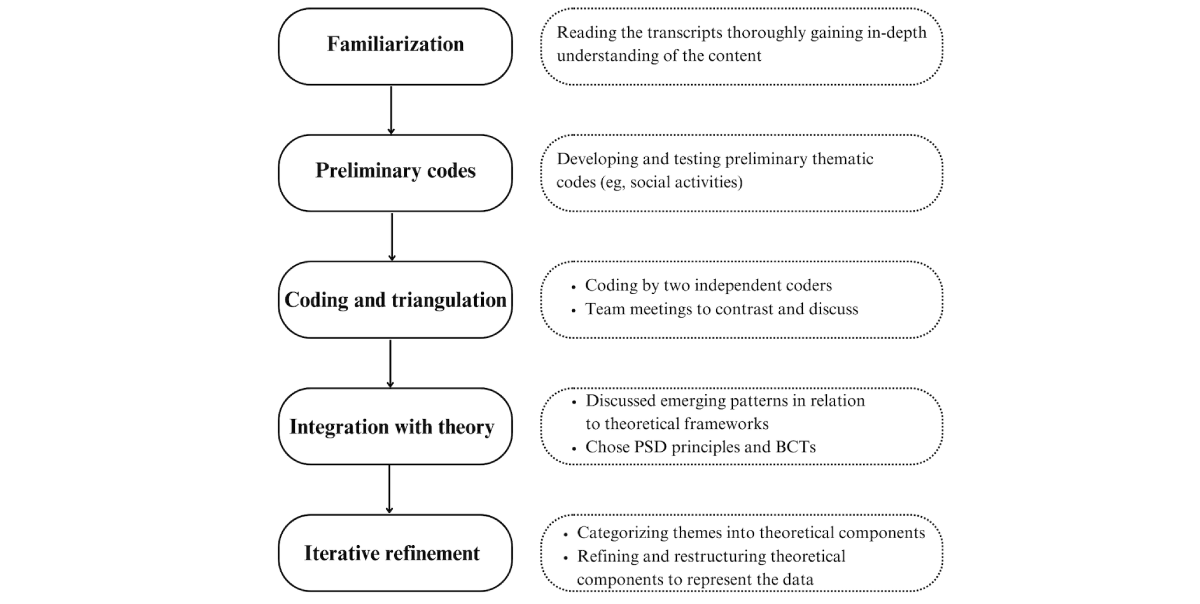
Steps in the abductive analysis process of focus group data on mHealth perspectives among retirement-age adults in Madrid, Spain (2022). This figure was developed by the authors based on guidelines for abductive qualitative analysis [31,32]. BCT: behavior change technique; PSD: persuasive systems design.

The analysis was facilitated using the software ATLAS.ti (Scientific Software Development GmbH). Three researchers participated in the coding process: the 2 focus group moderators (MSI and PCC) and an external researcher (RRR) who had not attended the focus groups to help ensure consistency in the coding strategy. To establish a common coding framework and ensure agreement, 3 researchers independently coded the first 2 transcripts. After reaching consistent interpretations and a shared understanding of the codebook, the remaining transcripts were coded independently by at least 2 researchers each. Discrepancies were resolved through discussion in all cases. The analysis followed an iterative process adopting an abductive approach [28-30]. This process consisted of 5 stages guided by the recommended steps for abductive analysis [31,32] (Figure 1):

(1) Familiarization: Reading the transcripts thoroughly to gain an in-depth understanding of the content.

(2) Preliminary coding: Developing and testing preliminary data-driven thematic codes (eg, preference for easy-to-use apps, low digital literacy as a barrier, preference for social/group activities, and accessible design).

(3) Coding and triangulation: At least 2 independent coders (MSI, PCC, and RRR) coded the material separately. Team meetings were held to compare and reconcile the coding and to ensure consistency across interpretations.

(4) Integration with theory: The team discussed emerging patterns in relation to existing theoretical frameworks. Different frameworks were considered for their relevance to designing mHealth interventions from a technology and behavior change perspective, so that the findings could be interpreted practically and inform future mHealth interventions. For behavior change, we examined frameworks like the theoretical domains framework [33], the COM-B model (Capability-Opportunity-Motivation-Behavior model) [34], and the behavior change techniques (BCTs) taxonomy V1 [35]. For technology-focused perspectives, we considered frameworks such as the technology acceptance model [36,37] and the persuasive systems design (PSD) framework [38].

Ultimately, we selected PSD and BCTs as the most appropriate frameworks for organizing our findings. PSD was chosen because most of the themes identified naturally aligned with its principles, providing a clear structure for translating user insights into design features. Although the technology acceptance model offers a useful framework for understanding predictors of mHealth engagement, such as perceived usefulness and ease of use, it does not provide guidance on the design elements that influence those perceptions, and was therefore less suitable for our purposes [36,37]. Regarding behavioral frameworks, while both the COM-B model and the theoretical domains framework are valuable for intervention development, they were not specific enough to capture the design-related preferences identified in our data. Instead, the BCTs Taxonomy allowed us to map participants’ preferences onto concrete psychological techniques that could be integrated into digital interventions.

Furthermore, PSD and BCT frameworks have been extensively used in previous qualitative studies and meta-analyses identifying effective mHealth components [39-42]. Moreover, recent research recommended considering both synergistically as these frameworks can be interrelated [43-45]. In this regard, we opted to report findings from both frameworks in an integrated manner, so we can understand which BCTs align with which PSD principles.

(5) Iterative refinement: In this phase, we revisited the initial data-driven codes to explore how they aligned with the selected frameworks’ components (PSD and BCTs). For example, participants’ interest in the organization of group activities was coded as “social activities,” which aligned with the PSD principle “social facilitation” and the BCT “social support (practical).” Similarly, preferences for personalized and age-appropriate recommendations (eg, recommended diets or goals depending on the user’s age, current activity) belonged in the “personalization” and “tailoring” principles. Furthermore, different BCTs were identified for supporting this (eg, goal setting, information about health and emotional consequences, graded tasks). This was conducted through a second coding strategy by at least 2 coders, resolving conflicts through discussion. This process enabled us to move from raw data to theory-informed interpretation while preserving the authenticity of participants’ perspectives. In fact, rather than imposing a pre-established theoretical framework, the data-driven analysis allowed for an iterative refinement, adjusting the theoretical categorization and structure according to the data and participants’ experiences. For example, different subprinciples were collated as these were discussed jointly (eg, tailoring and personalization). This reorganization was discussed in team meetings until an agreement was reached.

To address the potential risk of missing information inherent in deductive strategies [46], any theme that did not align with PSD principles or BCTs was still included in the results (ie, attitudes, barriers, and easy-to-use design), as it is recommended when conducting abductive analyses [28-30].

Additionally, when participants mentioned their use of mobile apps or commented on their own technological abilities, we noted this information to better contextualize their perspectives. Specifically, we documented the type of apps used (if mentioned) and made tentative observations about digital literacy levels when clearly reflected in the participants’ comments. For instance, we considered digital literacy to be low when participants expressed fear or difficulties in using apps, and high when they described frequent and confident use. This information, while not systematically collected, is reported in Table 1 for those participants whose input allowed for such interpretation.

Participants did not have the opportunity to review or provide feedback on these findings.

### Reporting

Selected quotes from participants were used to illustrate key themes and findings, and they are referenced throughout the manuscript. To maintain anonymity, limited demographic information is provided about the participants. Additionally, in Table S2 in Multimedia Appendix 1, quotes are presented in both the original language and their English translations. This study follows the COREQ (Consolidated Criteria for Reporting Qualitative Research) guidelines for reporting qualitative research (Table S3 in Multimedia Appendix 1) [47].

### Ethical Considerations

The study adheres to international ethical standards, including the Declaration of Helsinki [48]. Approval for the study was granted by the Comillas Pontifical University’s ethics committee (10/22-23). Informed consent was obtained from participants electronically when they registered through Microsoft Forms. The form also included contact information for the research team in case participants had any questions. At the start of each focus group, the moderators reiterated the study’s aims and confidentiality assurances and reminded participants that they could withdraw at any time. Consent was also obtained to record the audio of the sessions. No financial compensation was provided to participants for taking part in the study.

## Results

### Participants

The average age of participants was 61.5 years, and the majority were women (n=15). Table 1 provides further demographic information. Most participants were married (n=12), while 6 participants were either single or divorced, and one participant was widowed. A total of 9 participants explicitly mentioned using apps (for health purposes, banking, or learning languages), 4 participants referred to not using any, whereas 6 participants did not confirm or deny their use. Similarly, regarding digital literacy level, information to code this was only available for 7 participants (4 participants showed a low level, and 3 participants showed a high level).

### Findings Overview

Different PSD principles and BCTs were identified and discussed. Additionally, 2 themes emerged outside these categories: Attitudes and barriers, and easy-to-use design. An overview of the main findings and implications can be found in Table 2.

**Table 2:** Main themes, practical implications, and mapped persuasive systems design and numbered behavior change techniques (BCTs) from focus groups on mobile health (mHealth) among retirement-age adults (Madrid, Spain; 2022).

Themes and content			Description		Results and practical implications		BCTs^a^
**Persuasive systems design principles**							
	Primary Task Support	How the app can support users’ primary task through content and features.		Suggest meaningful, tailored, and progressive goals (eg, diet according to habits and age) Outline health and emotional consequences (eg, adrenaline after exercising) Preferred content: social/cultural activities, nutrition, physical activity, mental health, and relaxation. Ideas for substituting unhealthy snacks Self-monitoring without negative feedback		1.1,3. Goal setting 8.7. Graded tasks 5.1. Information about health consequences 5.6. Information about emotional consequences 8.2. Behavior substitution 2.3-4. Self-monitoring 2.2,7 Feedback (avoid negative)	
	Dialogue Support	Preferred communication and interaction methods		Positive feedback (messages) A moderate use of reminders Human-like interaction. An easy interactive voice response system Culturally appropriate communication (not anglicisms) Accessibility (sized text)		10.4. Social reward 7.1. Prompts/cues	
	System Credibility	Credibility and trust-building features		Avoid advertisements		—^b^	
	Social support	Social support and interaction features		Creation of groups for health behaviors (organize walk groups) Chat groups for sharing their experiences and barriers Video tutorials, but it is not the preferred format		3.1. Social support (unspecified) 3.3. Social support (practical) 4.1. Instructions on how to perform a behavior 6.1. Demonstration of the behavior	
**Emerging themes**							
	Attitudes and barriers	Perspectives on the use of technology for health purposes.		Open to using mHealth tools, even when initial attitudes are not positive. Low digital literacy as a barrier		—	
	Easy-to-use design	Preferences for intuitive designs		Intuitive, user-friendly, easy-to-use design Not excessively demanding or time-consuming. Clear symbols Short tutorials and onboarding guidance		4.1. Instructions on how to perform a behavior	

^a^Numbers correspond to the standardized numerical codes assigned to each technique within the BCT Taxonomy (v1) [35].

^b^Not applicable.

### Primary Task Support

#### Overview

It refers to how the app should support the user’s primary task. Participants identified several types of content they would like the app to provide, listed in order of priority: activities in their surroundings (social, educational, etc), nutrition, physical activity, mental health, and relaxation. This content was suggested to be delivered in different ways.

#### Tunneling and Reduction

Reduction refers to simplifying health behaviors so that they require less effort to initiate and maintain (eg, offering easy healthy recipes or snack substitutions), improving the benefit-cost ratio for users. Tunneling refers to guiding users through feasible options and providing means for actions that help them progress toward their goals (eg, suggesting nearby meaningful activities, thereby helping them identify concrete and achievable opportunities to engage in health-promoting behaviors). We grouped these 2 PSD principles together because participants often described them simultaneously when referring to what would help them take action using an mHealth tool.

Participants highlighted several system features that could both simplify health behaviors and suggest feasible opportunities to engage in them in daily life. For example, participants frequently mentioned they would like an app to provide information about social, educational, and cultural activities in their surroundings. For instance, P3 stated:

If it is feasible, <<Hey, tell me what activities are going on today in the neighborhood>>.

That would motivate me, that it offers me all kinds of alternatives, social, education or formative activities.

P9

Regarding nutritional information, participants requested tailored advice and diets based on their age group and personal habits (eg, usual physical activity). P2 explained: “a recommended diet tailored to my characteristics or to what I am doing at the moment if I am walking or not.” Furthermore, they would appreciate the app providing healthy recipes that are easy to cook and ideas for substituting unhealthy snacks for other healthier choices (BCT: behavior substitution).

Beyond nutrition, participants expressed interest in personalized challenges (feasible according to your goal and current status), memory exercises, relaxation, and ergonomic techniques. P10 noted:

Ergonomic techniques or techniques on how to activate your body that you can do at home (…) so that you can feel a little bit better in your body without the need of doing exercise.

#### Tailoring and Personalization

Participants agreed that the app’s content must be personalized to their individual needs (eg, diet/hydration requirements based on their current activity) or tailored to their age group (nutritional and physical activity goals recommended for their age). For instance, P2 emphasized:

Depending on that activity, it is advisable this quantity of nutrients or this quantity of x liquids, or I don’t know, it does not have to be only about food, so adequate hydration after the exercise you did.

Participants also wished to select their own goals, behaviors to monitor, and the frequency of notifications and reminders. Regarding goal setting (BCT), participants expressed that they would like these to be either tailored or personalized (based on their habits and age), emphasizing the significance of setting targets that are adequate and useful for them. For example, some participants outlined that they would appreciate the suggestion of meaningful goals tailored to their age. P13 shared:

It tells you to walk, but it should tell you how much you need to walk, I mean, not that I have walked 9400 steps or 10 kilometers, but if that is actually helpful or not (…) <<my objective would be to do this>>, even though there are many apps… but tailored to our age.

Participants suggested that motivating users to pursue health goals could involve highlighting relevant health (BCT: Health consequences) and emotional consequences (BCT: information about emotional consequences). As P13 described:

It should tell you the benefits, in the app, let you know the benefits of doing gymnastics, swimming, or whatever. It could tell you about adrenaline and that you are going to feel like a God.

Furthermore, participants expressed interest in goals that progress in difficulty based on their performance over time (BCT: Graded tasks). As mentioned, they also discussed the possibility of customizing the app’s complexity and settings to match the user’s level of technological knowledge.

#### Self-Monitoring

Several participants mentioned self-monitoring (BCT) as a valuable feature, allowing users to monitor steps, sleep time, water intake, or weight. For example, P7 stated:

Yes, I have this watch (smartwatch), and furthermore I record my weight, I weigh myself every day to check if I have lost a 100 grams <<laughs>>.

However, some participants expressed reluctance to monitor certain activities to avoid negative feedback (BCT). For example, one participant referred to avoiding monitoring sleep hours, acknowledging her insufficient sleep patterns:

I do not fill almost anything. Sleep hours? I do not fill sleep hours because I know that I sleep little. Objectively, I know what it is going to tell me…

P10

One participant admitted to inputting fake data when monitoring the behavior to accomplish the goals.

### Dialogue Support

#### Overview

Participants shared their views on how the app could implement effective computer-human dialogue to assist them in achieving their goals.

#### Praise and Rewards

Several participants expressed appreciation for receiving positive messages upon achieving their goals (BCT: Social reward). For instance, P7 stated:

When it says <<Look at that, you've already done the four days of exercise you had planned>> well, isn’t that great, you know?

However, as mentioned, some participants were reluctant to receive negative feedback to the point of avoiding self-monitoring or modifying their data to align with preset goals.

#### Reminders

Regarding reminders (BCT: prompts/cues), participants showed different perspectives on their usefulness and perceived value. At the same time, most found them useful for reminding them to carry out the behavior. Others did not consider reminders effective. Despite these differences, there was a general agreement that reminders should not be overly frequent or intrusive: P10 remarked, “It should not be overwhelming.” Participants suggested that allowing users to customize the frequency of reminders would be beneficial

#### Similarity and Social Role

Participants emphasized the importance of culturally appropriate language (avoiding anglicisms) within the app. P14 commented:

Because many times things get complicated, exactly, the terminology that is used. It is so easy to name things in Spanish. It is true; it really aggravates me; if we have such a wonderful vocabulary, a wonderful terminology, why do they have to complicate my life? I do not know what it means.

Some participants appreciated the idea of a more human-like interaction. For example, some would like the app to ask you how you are doing and to make you smile. In this regard, some suggested the possibility of an interactive voice response (IVR) as long as it does not make it harder to use it. P15 suggested:

I would suggest an app in which I can press something and it replies <<Hello, good morning, how are you today?>> (…) and please, sometimes, something for a smile, do not lose that sense of humor.

#### Liking

Participants valued certain features related to both aesthetics and functionality, such as an appealing logo and direct access to the main tools. Additionally, they emphasized the importance of accessibility and readability (eg, by using big enough letters and numbers). P13 explained:

Letters and numbers) they have to be really big, and they do not fit, I mean, that is a reality. In the numbers and letters, or you see them, or you go with your finger and press the one that is next to it.

### System Credibility Support

#### Overview

Participants shared their views on factors that contribute to an app’s credibility and trustworthiness.

#### Surface Credibility

Surface credibility refers to users’ initial judgments of an app’s credibility based on observable superficial cues, such as the presence and appropriateness of commercial content. Excessive, irrelevant, or intrusive advertisements can suggest commercial intentions, potentially reducing perceived trustworthiness of the app. In this regard, it was briefly mentioned that some participants would not use the app if it included advertisements.

### Social Support

#### Overview

The importance of social dimensions was consistently emphasized in all focus groups, emerging as a central aspect across themes. This emphasis underscores the value users place on fostering social connections and support.

#### Social Learning

Some participants expressed interest in learning by observing others performing specific behaviors. For example, exercise tutorial videos were seen as helpful tools (BCTs: Instructions on how to perform a behavior and demonstration of behavior). However, not all participants agreed. For example, one participant described feeling uncomfortable following exercise videos during the COVID-19 lockdown, even though her daughter encouraged her to use them. This highlights that video tutorials may not be universally preferred and that comfort with delivery format varies.

#### Social Facilitation

Participants expressed a strong desire for features that facilitate the creation of user groups within the app. These groups could serve as platforms for sharing experiences, like a “small WhatsApp” [P13], P12 explained: “Group therapies, each could tell their experiences or what it is they worry about at that moment” (BCT: Social support unspecified).

Others envisioned the possibility of organizing shared activities in the area, in this case, Madrid (eg, group walks; BCT: Social support practical). As P9 suggested:

If the app could facilitate the creation of a group and they say << let's go see Madrid de los Austrias (…)>> let’s see how many steps we make.

### Perspectives About eHealth: Attitudes and Barriers

Participants showed different perspectives and attitudes toward technology.

#### Positive and Negative Attitudes

Some participants exhibited a positive attitude towards using technology, such as smartphones or smartwatches, for managing their health. They particularly appreciated features like weight tracking and sleep regulation. For example, P2 monitors her weight daily on an app “I record it and you can check it, I see the graphics, it also regulates my sleep, I mean, the hours I sleep, and how much I walk. So… I do look at it and like it.” Conversely, some participants expressed reservations. For example, criticizing that technology is being overused, P3 stated that technology has facilitated duties so much that some common practices that we used to do before have now disappeared (ie, memorizing phone numbers or navigating routes without assistance), suggesting a negative impact of technology on our cognitive skills. However, they were still open to using these tools as long as they were easy to use.

#### Identified Barriers

Barriers to using technology are often related to participants’ perceived lack of skill and confidence. Some participants felt incapable of navigating technological devices (“I am terrible at it” [P17]), while others expressed fear and insecurity about making mistakes. These challenges reflect issues with digital literacy, encompassing both technical skills and the confidence to explore digital options without fear. P1 described this “fear”:

I am afraid of touching anywhere (…) They gave us 4 classes to learn how to handle mobile phones, but if anything pops up, I do not touch there because I do not know what will happen to me. Where I was taking the classes, they used to tell me <<it is okay, you can touch wherever, unless it is asking for your bank account, you can touch the rest, nothing is going to happen>> I say <<no, no, no, just in case I mess something up>>.

In this context, participants emphasized the importance of clear guidance on cybersecurity, such as knowing when not to click on suspicious links. Despite these challenges, most participants indicated that if the app were easy to use and offered valuable features, they would be open to using it.

### Easy-to-Use Design

Participants consistently emphasized the importance of simplicity, expressing a strong preference for an app that is intuitive and easy to navigate. P10 highlighted the importance of clear symbols: “A symbol is displayed that may be obvious for the developer, but maybe for you it is not obvious.”

Several participants agreed that the app should allow quick access to core features without requiring excessive time or effort to input data or learn how to use the system. They highlighted the need to avoid apps that are excessively demanding or time-consuming. Specifically, participants preferred not to spend significant time entering information or learning how to use the app. A common suggestion was the inclusion of a short tutorial for onboarding (BCT: Instructions on how to perform a behavior).

While there was a consensus on the importance of a simple and easy-to-use app, participants acknowledged that users’ needs might vary. P10 summarized this sentiment:

To the normal user, or to who is not going to use it for more than a couple of things, give him only that couple of things, and leave the rest to be like a step further. That is what I call a functional and simple app. The apps that achieve this that achieve that, allowing the geek to do geeky things and the regular person to do regular things.

## Discussion

### Principal Findings

#### Overview

This study provides valuable insights into the attitudes and experiences of Spanish retirement-age adults regarding mHealth interventions grounded in the PSD framework and identifying BCTs. The findings suggest that while older adults recognize the potential benefits of mHealth for promoting healthier lifestyles and are open to using them, several barriers hinder their adoption. The use of different PSD principles and BCTs (eg, social support) could enhance the persuasiveness and usage of these tools.

#### Primary Task Support

Participants discussed what content they would like to receive and how to receive it to facilitate promoting a healthy lifestyle. Their preferred content in order was social/cultural activities, nutrition, physical activity, mental health, and relaxation. This prioritization aligns with the biopsychosocial model of health, which underscores the need to address physical, mental, and social well-being holistically [49].

In addition, previous literature highlights users’ preference for a holistic understanding of health and well-being, incorporating stress and emotions rather than focusing solely on behaviors [44]. Consistently, they would like to be motivated not only by outlining the health consequences (BCT: 5.1. Information about health consequences) but also through emotional benefits (BCT: 5.6. Information about emotional consequences). While lifestyle interventions frequently use health consequences, emotional ones are usually ignored [39,50].

This preference aligns with self-determination theory [51], which can offer valuable insights on what type of consequences could be outlined to foster autonomous motivation, which is related to higher adherence [52,53]. In this regard, highlighting personally relevant consequences (eg, being independent for longer) or enjoyable ones (eg, fun, stress relief), rather than focusing only on health or disease prevention, may foster integrated/identified or intrinsic motivation, respectively [24]. Both forms of autonomous motivation could lead to higher engagement and maintenance of the target behavior [51,54]. Thus, outlining these consequences in an app could enhance its effectiveness in promoting health behaviors.

Regarding how they would like to receive the desired content, there was consensus on the need for tailoring and personalization based on users’ profiles and age groups, which was consistent with previous research [44,55]. Participants valued tailored diets (eg, what ingredients and liquids you should consume considering your usual activity), advice on behavior substitution (BCT: 8.2. Behavior substitution, such as ideas for substituting unhealthy snacks with healthier ones), and meaningful and progressive goals (BCTs: 1.1. Goal setting and 8.7. graded tasks: eg, start this week doing 1 hour of strength exercises, this improves your muscle function and will help you be strong and independent).

The importance of setting goals that are personally relevant to foster motivation also appeared in a previous qualitative study [44], and as already discussed, is essential to promote autonomous motivation and therefore long-term adherence [52,53]. Setting personally meaningful goals might be especially relevant in this population, as the socioemotional selectivity theory [56] proposes that, as individuals age, only activities that are meaningful and offer well-being are prioritized. In this regard, when an app suggests and sets a goal, identifying and outlining relevant consequences for the individual may improve the likelihood of its achievement.

One of the most appreciated features of mHealth was self-monitoring (BCTs: 2.3-4. Self-monitoring of behavior and of outcomes of behavior), as it allowed users to track their progress effectively. Participants noted that these tools provided valuable feedback and increased motivation. Although previous qualitative studies have not frequently highlighted this, this population widely uses self-monitoring apps [57]. Furthermore, self-monitoring has been proven to be an effective BCT in diverse meta-analyses [39,58], becoming an essential tool in any lifestyle intervention.

However, feedback valence played a critical role according to our participants. When negative feedback (BCTs: 2.2.7. Feedback on behavior or outcomes of behavior) was expected, some participants avoided introducing their data or even provided inaccurate information. This preference was found in previous qualitative studies: participants outlined the importance of feeling supported and receiving encouraging messages even on bad days, when they do not accomplish their goals [44].

This preference was further corroborated by a systematic review including qualitative and mixed studies [59], finding that positive and encouraging content was preferred, whereas negative feedback elicited negative feelings, discouraging app use. These reactions may reflect emotion regulation strategies. When faced with negative feedback, emotions such as anger or shame may arise, which some individuals attempt to avoid by disengaging from the source (eg, the app) or using distraction techniques [60].

Nevertheless, when looking at quantitative studies, there is controversy in the study of feedback: some studies find that feedback is useful in the promotion of health behavior, regardless of whether it is negative or positive [61], while others find that negative feedback could be less effective [62]. Furthermore, negative feedback can decrease self-efficacy [63] and make future efforts less likely.

Importantly, it is possible that negative feedback is more acceptable when it provides informative content or instructions that users were not previously aware of; for example, pointing out a lack of protein in a meal that was assumed to be balanced. In contrast, when feedback highlights known unhealthy behaviors, it may trigger feelings of guilt or shame, prompting users to avoid the app altogether as a means of emotional regulation.

In fact, other studies, mostly in the field of learning experiments, have found that how feedback is delivered is more important than whether it is positive or negative. For example, a meta-analysis showed that negative feedback decreased intrinsic motivation compared with positive feedback, but this effect was less pronounced when negative feedback included instructions for improvement [64]. Although this has been less explored in health behaviors, evidence suggests that gain-framed messages are overall more effective than loss-framed messages in promoting healthy behaviors [65,66], which could be considered when designing negative feedback messages in this context.

These findings offer a novel and valuable perspective on feedback in mHealth, highlighting the importance of avoiding strictly negative feedback and suggesting approaches to make it more acceptable and constructive. For example, combining negative feedback with actionable suggestions and using gain-framed messages. However, future studies should investigate these strategies in greater depth and determine how to deliver feedback effectively in mHealth interventions tailored to older adults.

#### Dialogue Support

One of the main ways in which an intervention is delivered through an app is through messages and dialogue. In fact, a meta-analysis found that in web-based interventions, a higher use of dialogue support elements was related to higher user adherence [42]. However, along with the Social support principle, these were the least implemented principles. Considering its importance, it is essential to understand how user-app communication should be established.

In this regard, our participants preferred a more human-like interaction, appreciating simple gestures such as asking “How are you doing today?” or receiving messages designed to make them smile or laugh. This reflects a preference for a social-oriented communication style, in contrast to a more task-oriented one, and is closely linked to the concept of social presence (the feeling of being with another [67,68]). Prior research has shown that social-oriented communication and higher perceived social presence foster trust, enjoyment, and emotional connection, which in turn increase engagement with digital tools [69,70].

However, communication preferences vary across individuals and cultural contexts. According to context theory [71], some people prefer high-context communication, which relies on implicit meanings, nonverbal cues, and social nuance, while others prefer low-context communication, characterized by direct, explicit messages and a focus on task efficiency. These preferences have been shown to moderate user engagement: for individuals who value relational and contextual cues (ie, high-context, socially oriented users), social presence enhances engagement; conversely, for task-oriented, low-context users, utilitarian features are more impactful [70].

Our findings suggest that participants in this Spanish sample tended to favor a socially oriented, high-context style of communication, which aligns with Spain’s classification as a high-context culture [72]. Recognizing and tailoring to these cultural and individual preferences is essential for effective mHealth design.

To support this type of communication, conversational artificial intelligence, whether through chatbots or IVR systems, could serve as a facilitator. Participants proposed the integration of simple interactive systems, such as IVRs. This aligns with prior findings showing that older adults often appreciate audio feedback features [55]. Furthermore, using a voice can increase social presence, and for those with preferences for social-oriented and high-context communication, it could be more appealing [73,74].

Participants also expressed a strong preference for avoiding technical terms and anglicisms, which was previously identified as a barrier [55].

These findings highlight the importance of culturally adapted communication, as preferences in terminology (eg, acceptance of anglicisms) and communication style (eg, high-context communication) can vary across cultures and generations. For instance, anglicisms might be more accepted by younger populations [75,76], and their usage differs depending on the language and cultural context [77]. Adopting a communication style that avoids unnecessary technical jargon and anglicisms, while fostering high-context communication and a strong sense of social presence, may better address the cultural and linguistic preferences of older Spanish adults.

Participants also mentioned accessibility as a critical design element. They noted that sometimes apps do not have an adequate letter-screen size ratio for this population, which appears as a usual barrier leading to difficulty in reading and interacting with the app effectively [55]. This aligns with existing guidelines recommending the use of large, legible fonts and touch-friendly interfaces to accommodate older users [78].

Regarding the messages and notifications, participants appreciated positive reinforcement, particularly receiving messages from the app that praised their achievements (BCT: 10.4. Social reward). This is consistent with previously found results outlining the importance of praise and reward messages to sustain user motivation [44,79].

Last, participants viewed reminders or prompts (BCT: 7.1. Prompts/cues) as useful tools to encourage behavior. This is consistent with prior literature, where prompts have been identified as facilitators in a systematic review of qualitative studies [55] and as an effective BCT for promoting physical activity in older adults [50]. However, participants emphasized that receiving too many notifications would be tiring, which could lead to alert fatigue [80], a commonly identified barrier [55]. To address this issue, participants suggested either a moderate use of reminders or allowing users to personalize the frequency and timing of notifications. This approach could prevent alert fatigue while maintaining the effectiveness of prompts to encourage health behaviors.

#### System Credibility

Participants mentioned the importance of an app free of publicity. Advertising in mHealth apps is often perceived as intrusive, which can erode user confidence and decrease engagement [81]. Ensuring a design free of advertisements could reinforce the app’s credibility and promote a sense of reliability among users.

Previous literature found concerns about security, data privacy, and trust in its management as a barrier to the adoption of eHealth tools [21,55]. However, in this study, such concerns were primarily associated with issues of digital literacy, derived from the fear and uncertainty of how to use apps safely. This highlights the critical need for clear communication about data security. A clear message about security and showing private handling of user data without advertisements will improve the perceived reliability of the app, particularly with cybersecurity concerns among aging people.

For instance, apps could include straightforward explanations of how personal data is protected, avoiding overly technical language to ensure comprehension among older users. Additionally, showcasing features such as encrypted data storage and the absence of third-party sharing could enhance the perceived reliability of the app, particularly for aging populations that are increasingly concerned about cybersecurity risks.

In addition to offering a secure, advertisement-free experience, system credibility can be further enhanced by making the app’s origin and affiliations transparent, such as clearly stating its development by reputable health institutions. Research has shown that perceived provenance (who developed the app and whether it is supported by trustworthy sources) has a strong influence on credibility among older adults [82]. Users may feel more confident when they know the app is endorsed by public health agencies, universities, or well-known hospitals. Displaying this information visually (eg, institutional logos, disclaimers, or mission statements) could reinforce legitimacy. Similarly, stating a credible source for the information provided could improve trust in the app [82,83]. For example, a common issue found in previous research was the low quality of information provided, without specifying a credible source or guaranteeing its correctness [82,83].

mHealth developers can reduce trust-related barriers and foster greater acceptance of these tools among older adults by addressing these issues directly: communicating clearly, eliminating intrusive advertising, highlighting institutional credibility, including high-quality information from credible sources, and visibly signaling data protection.

#### Social Support

Social support and involvement emerged as one of the most mentioned and valued topics, with participants discussing its influence in different ways and through multiple approaches. This is consistent with previous literature, as social support has been systematically found as an essential component when studying the acceptance, use, and adherence to eHealth tools in older adults [23,55,84,85]. Social support contributes not only to increased engagement but also to better health outcomes, as it fosters a sense of community and shared motivation [39]. Especially, external social support from family members, caregivers, or health care providers has been found to predict greater use and adherence to eHealth technologies [23,55,84,85]. Despite its recognized importance, social support is usually the least implemented principle in mHealth, along with dialogue support [41].

In this study, participants expressed a strong interest in features that facilitated social interaction within the app. First, they suggested the inclusion of tools for organizing or recommending group activities (BCT: 3.3. Social support practical; eg, meeting to get to know a part of the city and count the steps). Second, they appreciated the idea of group chats where users could share their experiences, difficulties, and barriers, supporting each other (BCT: 3.1. Social support unspecified). These preferences indicate a clear demand for mechanisms that foster peer-to-peer interaction and mutual encouragement. Interestingly, these findings align with prior studies that have identified online support groups as a desirable feature for older adults [44,84]. Including these features could facilitate engagement, enjoyment, and also make health behaviors (eg, walking) an opportunity for social affiliation (an intrinsic goal from the self-determination theory), which could lead to autonomous motivation and better adherence to the behavior.

Participants also mentioned that instructional videos could be useful for demonstrating behaviors (BCT: 6.1. Demonstration of the behavior, 4.1. Instructions on how to perform a behavior) when other options are unavailable. However, some participants were reluctant to follow such videos, suggesting that their preferences may vary based on individual comfort levels with this format. This variability aligns with previous research indicating that while many older adults exhibit positive attitudes toward video-based interventions and adhere to this format [86], others may prefer alternative methods of instruction, such as written guides or interactive tutorials. For instance, the Institute on Aging [87] highlighted that older adults often favor written materials for their clarity and accessibility, as these formats are perceived as straightforward and less overwhelming. Additionally, interactive tutorials were shown to enhance engagement by allowing users to learn at their own pace, accommodating varying levels of digital literacy and familiarity with technology. These findings emphasize the importance of offering diverse instructional formats to meet the heterogeneous preferences and needs of older adults effectively.

Overall, these results point out the importance of integrating social support features into mHealth tools for older adults. By enabling social interaction and offering multiple instructional formats, developers can address individual differences and enhance both engagement and adherence.

#### Attitudes, Barriers, and Design Preferences

Similar to previous research, our findings indicate an overall positive attitude toward the use of eHealth and mHealth, as well as openness to use them [88]. However, certain concerns and barriers influencing the use of these tools arose, such as (1) concern for technology dependence and (2) low digital literacy.

Regarding technology dependence, participants criticized that tasks previously performed manually (such as route planning) are now often outsourced to technology, reflecting that excessive reliance on digital tools can lead to a decline in memory retention and navigational abilities. Studies partially support this concern, showing that overly relying on technology can lead to negative cognitive consequences, such as reduced memory retention and attention span. However, recent studies have shown that technology can also improve cognitive functioning, potentially enhancing memory, attention, and executive function when used appropriately [89-91]. Understanding this hesitance and concerns is critical for eHealth developers, who could explicitly address them by incorporating features designed to counteract cognitive decline. For instance, integrating cognitive training tasks or memory-enhancing games could encourage users to perceive technology as a tool for improving, rather than replacing, their cognitive skills. Furthermore, the use of these tools could also be framed as a learning challenge that could be cognitively stimulating.

Low digital literacy appeared in terms of participants’ perceived lack of competence and fear of severe consequences if used incorrectly. This is consistent with prior reviews, indicating that older adults often experience low self-efficacy, lack of confidence, and a general aversion or fear toward technology [55]. These challenges lead to a cycle of avoidance and missed opportunities to benefit from digital tools.

Participants identified several design elements that could help overcome this barrier. They emphasized the importance of a user-friendly and intuitive app design that minimizes cognitive and technical demands; an app that is not excessively demanding or time-consuming. In fact, it has been found that information or system features overload in mHealth relates to higher resistance and reluctance to use these tools in older adults [80]. Thus, reducing this cognitive load is essential to foster engagement and minimize dropout rates among older users [78].

Participants suggested additional actionable ways to improve usability, such as incorporating clear, universally understandable symbols and short tutorials for onboarding. How to design an adequate tutorial would be another key point, for which different guidelines for older adults can be followed (eg, avoid cognitive low and burden on working memory) [92]. Further training on technology might also be useful to address this barrier. These features can ease the learning process and enhance users’ confidence in navigating the app. This aligns with previously identified facilitators in the literature, which recommend prioritizing simplicity and providing step-by-step guidance to improve user experience [55,78].

### Limitations

This study provides valuable insights into the perspectives of Spanish retirement-age adults regarding mHealth interventions; however, several limitations should be considered when interpreting the findings.

First, although the number of focus groups aligns with prior recommendations for qualitative research [27], data saturation was not formally assessed and therefore cannot be confirmed. Thus, it is possible that some influencing factors were not identified. Second, the sample size was relatively small (n=19) and predominantly female. Although the initial recruitment strategy did not target any specific gender, once a gender imbalance became evident, additional efforts were made to recruit male participants. Despite these efforts, participation remained predominantly female (15/19, 79%). This limitation, common in research [93], and particularly with this population [94,95], may limit the generalizability of the results and highlights the need for more targeted strategies to engage men in future research. Therefore, while the findings shed light on important themes, a larger and more diverse sample, including more male participants, could provide a broader understanding of mHealth preferences among this population.

Additionally, all participants were recruited from Madrid, an urban area, which may not fully capture the experiences and needs of individuals from rural regions with different technological adoption rates and cultural contexts. Moreover, while information on participants’ employment (current or prior to retirement) was collected as an indirect indicator of socioeconomic status, other demographic variables such as education level and income were not systematically assessed. Expanding the geographic scope and using a more comprehensive demographic assessment in future studies could enhance the contextualization of the findings.

Additionally, while the PSD and BCT frameworks provided a valuable structure for organizing and interpreting the data, they present certain limitations. Specifically, some salient themes that emerged (such as attitudes, barriers, and easy-to-use design) did not clearly align with any of the pre-established categories within these models. This suggests that although these frameworks are useful for identifying actionable techniques and persuasive strategies, they may not fully capture all relevant aspects for older adult users. Future research could consider complementing these models with additional theoretical perspectives to ensure a more holistic understanding of user needs.

Finally, even though several preferences are highlighted, these might not always be feasible for app design and can actually differ across individual characteristics such as digital literacy or prior technology use. While we report general information on these variables when available to contextualize participants’ quotes, the small sample size and qualitative design do not allow for meaningful subgroup comparisons. Future quantitative studies could build upon these findings to explore, for example, whether preferences for features such as human-like interaction or group-based activities differ according to digital literacy level or other relevant variables. Such studies could further inform the personalization of mHealth tools and contribute to reducing digital health disparities in this population.

### Conclusions

This study explores retirement-age adults’ perspectives on mHealth interventions, revealing key opportunities and challenges in their design and implementation. Tailoring mHealth tools to the needs of older adults, guided by the PSD framework and BCTs could improve these interventions.

Participants expressed a willingness to adopt mHealth technologies, favoring tools that are user-friendly, intuitive, and personalized. Preferred BCTs included goal setting, self-monitoring, and social rewards, alongside features fostering social connections through group activities and support networks. The findings also emphasize the importance of culturally and age-appropriate design, such as avoiding technical jargon, using accessible interfaces, and human-like communication.

Despite positive attitudes, significant barriers were identified, including low digital literacy, fear of misuse, and concerns about overdependence on technology and its potential negative impact on cognitive functioning. Incorporating onboarding tutorials, clear cybersecurity guidance, and cognitive support features could address these challenges. By integrating user-centered design and addressing these barriers, developers can create empowering tools that promote healthier aging and enhance quality of life in older adults.

## Data Availability

The datasets generated or analyzed during this study are available from the corresponding author on reasonable request.

## Appendix

Multimedia Appendix 1 Focus group interview guide, original Spanish quotations with English translations, and COREQ checklist.

## Abbreviations

BCT: behavior change technique
COM-B: Capability-Opportunity-Motivation-Behavior
COREQ: Consolidated Criteria for Reporting Qualitative Research
IVR: interactive voice response
mHealth: mobile health
PSD: persuasive systems design
