# Supplementary material for: Tailoring mHealth for Healthy Aging: Focus Group Study With Retirement-Age Adults
Source: JMIR Mhealth Uhealth. 2025 Dec 15;13:e70051. doi: 10.2196/70051 (PMC12750076; doi:10.2196/70051)
Supplement: Multimedia Appendix 1 [file mhealth_v13i1e70051_app1.docx]

**Supplementary Material**

**Table S1.** Focus group discussion guide

| **Introduction** |
| --- |
| - Introduction of the SO-NUTS project and the goals of this study - Voluntary participation, right to leave the study anytime - Protection of the data and confidentiality - Permission to record the audio |
| **Section 1: facilitators and barriers for health behaviors** |
| - This section explored retirement-age adults’ perceptions of healthy lifestyles, motivations, barriers, and facilitators related to behavior change. These questions were analyzed and reported in a previously published study [24] and therefore were not included in the present analysis. |
| **Section 2: perspectives and usage of digital tools and m-health** |
| 1. Nowadays we all assume that everyone has a smartphone, do you have one? 2. Have you ever used a health app (step counter, how you slept)? 3. Would you like to use an app that can help you in this way? 4. What characteristics should such an app have to be useful for you? |

**Table S2.** Original quotes and their translation

| **P** | **Original** | **Translation** |
| --- | --- | --- |
| P2 | *“lo apunto y vas viendo, veo la gráfica también me regula el sueño o sea las horas que duermo, las que no y lo que ando. Entonces, bueno, pues sí que lo, sí que lo miro y me gusta”* | *“I record it and you can check it, I see the graphics, it also regulates my sleep, I mean, the hours I sleep, and how much I walk. So… I do look at it and like it”.* |
| P17 | *Soy una negada”* | *“I am terrible at it”* |
| P1 | *“Me da miedo tocar donde sea (…) han dado 4 clases para que aprendamos un poco a manejar los móviles, pero claro, yo ahora mismo me pasa cualquier cosa, me viene cualquier cosa y es que no le tocó, ahí no toco, porque no sé lo que me va a pasar. Donde está ahora que me daban clases decían “<<que no pasa nada, tú puedes tocar donde… menos donde te pidan tu número del banco lo demás puedes tocar todo, que no te va a pasar nada>> digo <<no, no, no, no sea que líe alguna>>”* | *“I am afraid of touching anywhere (…) They gave us 4 classes to learn how to handle mobile phones, but if anything happens now, anything pops up, I do not touch, I do not touch there because I do not know what will happen to me. Where I was taking the classes, they used to tell me <<it is okay, you can touch wherever, unless it is asking for your bank account, you can touch the rest, nothing is going to happen>> I say <<no, no, no, just in case I mess something up>>”.* |
| P10 | *“te ponen un símbolo que a lo mejor para el desarrollador es muy evidente, pero que a lo mejor para ti no es evidente”* | *“A symbol is displayed that may be obvious for the developer, but maybe for you it is not obvious”.* |
| P10 | *“Entonces, al usuario normalito y corriente o que no va usar más que para cuatro cosas, darle sólo esas cuatro cosas y que el otro sea como un paso, además. Eso es un poco lo que yo llamo una aplicación funcional y sencilla. Las aplicaciones que consiguen eso, que consiguen que el friki pueda hacer las cosas de friki, que el normal haga las normales”* | *“To the normal user, or to who is not going to use it for more than a couple of things, give him only that couple of things, and leave the rest to be like a step further. That is what I call a functional and simple app. The apps that achieve this that achieve that, allowing the geek to do geeky things and the regular person to do regular things”.* |
| P3 | *“si es que es factible, oye, <<dime qué actividades hay hoy en el barrio>>”* | *“If it is feasible, <<Hey, tell me what activities are going on today in the neighborhood>>.”* |
| P9 | *“eso me motivaría, que ofrezca alternativas de todo tipo, sociales, de educación, formativas”* | *“That would motivate me, that it offers me all kinds of alternatives, social, education or formative activities.”* |
| P2 | *“me viniera eh… una dieta recomendada eh…para mis características o para lo que estoy haciendo en ese momento, pues si ando o no ando”* | *“a recommended diet tailored to my characteristics or to what I am doing at the moment if I am walking or not.”* |
| P10 | *“técnicas ergonómicas o cómo activar el cuerpo que puedan hacerse en casa (…) para estar un poco mejor en el cuerpo sin necesidad de hacer deporte”* | *“Ergonomic techniques or techniques on how to activate your body that you can do at home (…) so that you can feel a little bit better in your body without the need of doing exercise”.* |
| P2 | *“pues dependiendo de esa actividad, pues es recomendable esta cantidad de nutrientes o esta cantidad de beber x líquidos o no sé, porque no tiene por qué ser solo comida, pues una hidratación adecuada para el ejercicio que has realizado”* | *“Depending on that activity, it is advisable this quantity of nutrients or this quantity of x liquids, or I don’t know, it does not have to be only about food, so adequate hydration after the exercise you did”* |
| P13 | *“si te dice que camines, pero que te diga qué tienes que caminar, quiero decir, a ver si me explico, no que he hecho 9400 pasos o que he hecho 10 kilómetros, que, si realmente eso me resulta útil o no (…) «Mi objetivo sería hacer esto», aunque ya hay muchas apps que lo… pero bueno, aplicado a nuestra edad”* | *“It tells you to walk, but it should tell you how much you need to walk, I mean, not that I have walked 9400 steps or 10 kilometers, but if that is actually helpful or not (…) <<my objective would be to do this>>, even though there are many apps… but tailored to our age”* |
| P13 | *“* | *“it should tell you the benefits, in the app, let you know the benefits of doing gymnastics, swimming, or whatever. It could tell you about adrenaline and that you are going to feel like a God”.* |
| P7 | *“sí, yo tengo el reloj este y además me apunto el peso, yo me peso todos los días, para ver si he bajado 100 gramos <<se ríe>>”* | *“Yes, I have this watch (smartwatch), and furthermore I record my weight, I weigh myself every day to check if I have lost a 100 grams <<laughs>>”* |
| P10 | *“yo no lo relleno prácticamente nada. ¿Las horas de sueño? Las horas de sueño no las relleno pues porque sé que duermo poco <<se ríe>> objetivamente, y sé que me va a decir que…”* | *“I do not fill almost anything. Sleep hours? I do not fill sleep hours because I know that I sleep little. Objectively, I know what it is going to tell me…”* |
| P7 | *“cuando dice, <<mira qué bien, ya has hecho los cuatro días que tenían previsto de ejercicio>> pues mira qué bien ¿sabes?”* | *“When it says <<Look at that, you've already done the four days of exercise you had planned>> well, isn’t that great, you know?”* |
| P8 | *“si encima te está diciendo, recordándote “tienes que ponerte” Sí, sí ayuda.”* | *“If besides it is telling you, reminding you <<you have to do it>>, yes, yes it helps”* |
| P17 | *“«Descárgate una aplicación que te recuerde». Y entonces me descargué la aplicación y es que no le hago caso, sigo sin beber agua”* | *“<<download an app that reminds you>>, so I downloaded the app, and I ignore it, I still do not drink water.”* |
| P10 | *“que no te agobie en exceso”* | *“It should not be overwhelming.”* |
| P14 | *“Porque muchas veces complican las cosas, exactamente, la terminología que se usa. Cuando es tan fácil llamarlo en castellano las cosas. Es que, de verdad, yo a mí eso me pone también negra, porque si tenemos un vocabulario estupendo, tenemos una terminología estupenda, por qué me tienen que complicar la vida de esta manera, que no sé lo que quiere decir”* | *“Because many times things get complicated, exactly, the terminology that is used. It is so easy to name things in Spanish. It is true; it really aggravates me; if we have such a wonderful vocabulary, a wonderful terminology, why do they have to complicate my life? I do not know what it means”.* |
| P15 | *“Yo plantearía una app en la que pulses algo y diga «Hola, buenos días, ¿qué tal hoy?» (…) Y por favor, en algún momento algo para alguna sonrisa, que no perdamos el sentido del humor.”* | *“I would suggest an app in which I can press something and it replies <<Hello, good morning, how are you today?>> (…) and please, sometimes, something for a smile, do not lose that sense of humor”.* |
| P13 | *“Tienen que ser muy grandes los números y en un móvil no entran, quiero decir, que eso es una realidad. En los números y en las letras. El ver, o vas con el dedo y le das al de al lado”* | *“(Letters and numbers) they have to be really big, and they do not fit, I mean, that is a reality. In the numbers and letters, or you see them, or you go with your finger and press the one that is next to it”* |
| P12 | *“Terapias de grupo, que cada uno cuente sus vivencias o qué le preocupa en ese momento”* | *“Group therapies, each could tell their experiences or what it is they worry about at that moment”.* |
| P13 | *“* | *“small WhatsApp”* |
| P9 | *“* | *“if the app could facilitate the creation of a group and they say << let's go see Madrid de los Austrias (…)>> let’s see how many steps we make”.* |

**Note.** P: Participants’ ID

**Table S3.** Consolidated criteria for reporting qualitative studies (COREQ): 32-item checklist

| **No. Item** | **Guide questions/description** | **Reported on Page #** |
| --- | --- | --- |
| **Domain 1: Research team and reﬂexivity** |  |  |
| *Personal Characteristics* |  |  |
| 1. Inter viewer/facilitator | Which author/s conducted the interview or focus group? | Page 3 “Procedure” |
| 2. Credentials | What were the researcher’s credentials? E.g. PhD, MD | Page 3 “Procedure” |
| 3. Occupation | What was their occupation at the time of the study? | Page 3 “Procedure” |
| 4. Gender | Was the researcher male or female? | Page 3 “Procedure” |
| 5. Experience and training | What experience or training did the researcher have? | Page 3 “Procedure” |
| *Relationship with participants* |  |  |
| 6. Relationship established | Was a relationship established prior to study commencement? | Page 3 “Procedure” |
| 7. Participant knowledge of the interviewer | What did the participants know about the researcher? e.g. personal goals, reasons for doing the research | Page 3 “Procedure” |
| 8. Interviewer characteristics | What characteristics were reported about the inter viewer/facilitator? e.g. Bias, assumptions, reasons and interests in the research topic | Page 3 “Procedure” |

| **Domain 2: study design** |  |  |
| --- | --- | --- |
| *Theoretical framework* |  |  |
| 9. Methodological orientation and Theory | What methodological orientation was stated to underpin the study? e.g. grounded theory, discourse analysis, ethnography, phenomenology, content analysis | Page 3-4 “Data Analysis” |
| *Participant selection* |  |  |
| 10. Sampling | How were participants selected? e.g. purposive, convenience, consecutive, snowball | Page 2 “Participants” |
| 11. Method of approach | How were participants approached? e.g. face-to-face, telephone, mail, email | Page 2 “Participants” |
| 12. Sample size | How many participants were in the study? | Page 2 “Participants” |
| 13. Non-participation | How many people refused to participate or dropped out? Reasons? | Page 2 “Participants” |
| *Setting* |  |  |
| 14. Setting of data collection | Where was the data collected? e.g. home, clinic, workplace | Page 3 “Procedure” |
| 15. Presence of non-participants | Was anyone else present besides the participants and researchers? | Page 3 “Procedure” |
| 16. Description of sample | What are the important characteristics of the sample? e.g. demographic data, date | Page 2 “Participants”; Table 1 |
| *Data collection* |  |  |
| 17. Interview guide | Were questions, prompts, guides provided by the authors? Was it pilot tested? | Page 3 “Data Collection” |
| 18. Repeat interviews | Were repeat interviews carried out? If yes, how many? | Page 3 “Procedure” |
| 19. Audio/visual recording | Did the research use audio or visual recording to collect the data? | Page 3 “Data Collection” |
| 20. Field notes | Were ﬁeld notes made during and/or after the interview or focus group? | Page 3 “Data Collection” |
| 21. Duration | What was the duration of the interviews or focus group? | Page 3 “Data Collection” |
| 22. Data saturation | Was data saturation discussed? | Page 3 “Procedure” |
| 23. Transcripts returned | Were transcripts returned to participants for comment and/or correction? | Page 3 “Data Collection” |
| **Domain 3: analysis and ﬁndings** |  |  |
| *Data analysis* |  |  |
| 24. Number of data coders | How many data coders coded the data? | Page 3-4 “Data analysis” |
| 25. Description of the coding tree | Did authors provide a description of the coding tree? | Page 3-4 “Data analysis” |
| 26. Derivation of themes | Were themes identiﬁed in advance or derived from the data? | Page 3-4 “Data analysis” |
| 27. Software | What software, if applicable, was used to manage the data? | Page 3-4 “Data analysis” |
| 28. Participant checking | Did participants provide feedback on the ﬁndings? | Page 3 “Data Collection” |
| *Reporting* |  |  |
| 29. Quotations presented | Were participant quotations presented to illustrate the themes/ﬁndings? Was each quotation identiﬁed? e.g. participant number | Page 5 “Reporting” |
| 30. Data and ﬁndings consistent | Was there consistency between the data presented and the ﬁndings? | Page 5 “Reporting” |
| 31. Clarity of major themes | Were major themes clearly presented in the ﬁndings? | Page 5 “Reporting” |
| 32. Clarity of minor themes | Is there a description of diverse cases or discussion of minor themes? | Page 5 “Reporting” |
